# Development of a multilayer fetal membrane material model calibrated using bulge inflation mechanical tests

**DOI:** 10.1016/j.jmbbm.2023.106344

**Published:** 2023-12-28

**Authors:** Daniel S. Fidalgo, Kayvan Samimi, Michelle L. Oyen, Melissa C. Skala, Renato M.N. Jorge, Marco P.L. Parente, Ewelina Malanowska, Dulce A. Oliveira, Kristin M. Myers

**Affiliations:** a Institute of Science and Innovation in Mechanical and Industrial Engineering (INEGI), R. Dr. Roberto Frias 400, 4200-465, Porto, Portugal; b Mechanical Department (DEMec), Faculty of Engineering of University of Porto (FEUP), Rua Dr. Roberto Frias, 4200-465, Porto, Portugal; c Morgridge Institute for Research, Madison, WI, USA; d Department of Biomedical Engineering, Washington University in St. Louis, St. Louis, MO, USA; e Department of Biomedical Engineering, University of Wisconsin, Madison, WI, USA; f Department of Gynaecology, Endocrinology and Gynaecologic Oncology, Pomeranian Medical University, Szczecin, Poland; g Department of Mechanical Engineering - Columbia University, New York, NY 10027, USA

**Keywords:** Fetal membrane, Amnion, Chorion, Inflation test, Constitutive model, Numerical simulation

## Abstract

The fetal membranes are an essential mechanical structure for pregnancy, protecting the developing fetus in an amniotic fluid environment and rupturing before birth. In cooperation with the cervix and the uterus, the fetal membranes support the mechanical loads of pregnancy. Structurally, the fetal membranes comprise two main layers: the amnion and the chorion. The mechanical characterization of each layer is crucial to understanding how each layer contributes to the structural performance of the whole membrane. The *in-vivo* mechanical loading of the fetal membranes and the amount of tissue stress generated in each layer throughout gestation remains poorly understood, as it is difficult to perform direct measurements on pregnant patients. Finite element analysis of pregnancy offers a computational method to explore how anatomical and tissue remodeling factors influence the load-sharing of the uterus, cervix, and fetal membranes. To aid in the formulation of such computational models of pregnancy, this work develops a fiber-based multilayer fetal membrane model that captures its response to previously published bulge inflation loading data. First, material models for the amnion, chorion, and maternal decidua are formulated, informed, and validated by published data. Then, the behavior of the fetal membrane as a layered structure was analyzed, focusing on the respective stress distribution and thickness variation in each layer. The layered computational model captures the overall behavior of the fetal membranes, with the amnion being the mechanically dominant layer. The inclusion of fibers in the amnion material model is an important factor in obtaining reliable fetal membrane behavior according to the experimental dataset. These results highlight the potential of this layered model to be integrated into larger biomechanical models of the gravid uterus and cervix to study the mechanical mechanisms of preterm birth.

## 1. Introduction

The fetal membranes are a complex biological structure that surrounds and protects the fetus, serving as a mechanical and immunological barrier throughout pregnancy (Bryant-Greenwood, 1998; Jabareen et al., 2009). It is a multilayered structure undergoing complex microstructural changes by the end of pregnancy (McLaren et al., 1999), contributing to tissue weakening in preparation for birth (Oyen et al., 2006), and undergoing an intense growth and deformation process to adapt to the different stages of pregnancy (Joyce et al., 2016). At term, the following dynamic events occur: cervical deformation, rupture of membranes, and uterine contractions (Vink and Feltovich, 2016). These phenomena involve coordinated responses from the fetal membrane, the cervix, and the uterus, whose interactions are not completely understood (Vink and Feltovich, 2016). The rupture of the fetal membranes (ROM) is an integral part of vaginal delivery but can lead to serious maternal and fetal health problems when it occurs before term (Devlieger et al., 2006). Approximately 3% of all pregnancies worldwide suffer a membrane rupture earlier than 37 weeks gestational age, called preterm pre-labor rupture of the fetal membranes (PPROM) (Mercer, 2010).

Generally, the membranes comprise two main layers, amnion and chorion, whose microstructures differ significantly from each other (Helmig et al., 1993). The amnion is the inner layer of the membranes facing the amniotic fluid, while the chorion is the outer layer contacting the uterine wall (Ilancheran et al., 2009; Zhang et al., 2021). The amnion layer, a fibrous time-dependent material, is the mechanically dominant layer of the fetal membranes (Buerzle and Mazza, 2013; Oyen et al., 2004, 2006). Randomly oriented collagen fibers dominate the material response to tensile loading. Hence, a constitutive material model capturing fiber stretching and rotation mechanisms helps understand how the fetal membranes help load-share with the uterus and cervix (Buerzle and Mazza, 2013). The amnion is thinner, stiffer, and stronger than the compliant and more extensible chorion (Buerzle et al., 2012; Buerzle and Mazza, 2013; Oyen et al., 2004). The amnion and chorion are separated by a spongy layer, which comprises proteoglycans and glycoproteins, along with collagen of type III, and the spongy layer interface allows the amnion to slide on the underlying chorion (Joyce et al., 2009; Menon and Richardson, 2017).

The decidua, a maternal layer adjacent to the chorion, plays an essential role in the mechanics of the fetal membranes (Tahan and Tahan, 2014). This layer results from the uterine endometrium’s differentiation process under the influence of progesterone (Abbas et al., 2019). It is known that the decidua shares a close anatomical relationship with the chorion (chorio-decidua layer), which might suggest that decidual changes may be associated with chorioamnion membrane alterations (Malak and Bell, 1994). The development of the decidual layer and the chorion is synchronized from the earliest phase of implantation, and the smooth chorion becomes fused with the decidua (Ogura et al., 2017).

Foundational mechanical tests have been performed on the fetal membranes, often characterizing individual layers from at-term delivered samples. Oxlund et al. designed a method to retrieve uniaxial data concerning the mechanical properties of the fetal membranes (Oxlund et al., 1990). Helmig et al. analyzed whether the mechanical properties of the fetal membranes changed during late pregnancy and labor, concluding that pronounced changes were induced in the amnion and chorion mechanical properties (Helmig et al., 1993). Oyen et al. investigated the soft-tissue time-dependent behavior, focusing on the amnion sublayer, and the differences between the amnion and the chorion in terms of failure properties, analyzing the biaxial puncture failure of intact chorioamnion, and separated amnion and chorion (Oyen et al., 2004, 2006). Oyen et al. also performed uniaxial and planar biaxial mechanical tests to determine the amnion layer’s nonlinear and time-dependent mechanical response (Oyen et al., 2005). Verbruggen et al. developed linear elastic finite element models of fetal membranes to determine the mechanical role of the amnion and the chorion layers, and to simulate the uterine environment in the context of term gestation and PPROM (Verbruggen et al., 2017). However, the chorioamnion – and the amnion in particular – is strongly nonlinear in its mechanical response (Oxlund et al., 1990; Oyen et al., 2005), so the Verbruggen study is limited in its applicability.

This collection of mechanical tests and models highlights the complex material behavior of fetal membranes. It motivates a need to develop a layered model to capture its robust mechanical function. Understating the complex interaction between the membranes, the cervix, and the uterus at term or preterm requires accurate modeling of the fetal membrane, especially due to the amnion’s anisotropic nonlinear behavior, which dominates the membrane’s mechanical response. Therefore, this work develops a multilayer fetal membrane biomechanical model capturing the physiological response of fetal membranes within the uterine environment. This model represents an improvement over the existing ones by considering the amnion as an anisotropic nonlinear material. The validation of the model is performed by comparing the inflation apex displacement of the model with an inflation mechanical dataset (Samimi et al., 2023), and comparing the numerical maximum principal stress values in the amnion and chorion layers with the respective physiological ranges reported in the literature.

## 2. Materials and methods

This work is divided into three main parts: (i) mechanical characterization of the amnion, chorion, and decidua, (ii) development of a multilayer fetal membrane model, and (iii) validation of the multilayer model. In part (i), the constitutive model used to characterize the amnion is presented, and the respective material parameters are; the chorion and decidua are characterized using a linear elastic constitutive model with parameters retrieved from the literature. In part (ii), a multilayer fetal membrane model comprising the fetal amnion, chorion, and maternal decidua is developed, applying the constitutive characterization presented in part (i). Finally, in part (iii), the layered model is validated by comparing the outcomes of an inflation numerical simulation (apex displacement, maximum principal stress, and thickness variation), with experimental data and information reported in the literature.

### 2.1. Amnion, chorion, and decidua models

#### 2.1.1. Amnion constitutive model formulation

In this work, a modified version of the Buerzle-Mazza formulation (Buerzle and Mazza, 2013) developed by Mauri and Ehret (Mauri et al., 2016) was considered to characterize the mechanical response of the amnion (Mauri et al., 2016). The strain energy function, Ψ, represents the strain energy per unit reference volume and accounts for the stretch of the single families of fibers (Buerzle and Mazza, 2013; Mauri et al., 2016):

(2.1) $$\Psi =\frac{\mu_{0}}{2q}\left[e^{qg}-1\right]$$

where $\mu_{0}$ is a material constant having the units of stress and q is a dimensionless constant that controls the nonlinearity of the moduli for the different stress responses in the composite structure (Rubin and Bodner, 2002).

The function g is given by the sum of the compressible neo-Hookean material $g_{2}$, and the fiber strain energy $g_{3}$ (Buerzle and Mazza, 2013; Mauri et al., 2016):

(2.2) $$g=g_{2}\left(I_{1},J\right)+g_{3}\left(\lambda_{i}\right)$$

(2.3) $$g_{2}=m_{2}\left(I_{1}-3\right)+\frac{m_{2}}{m_{5}}\left(J^{-2m_{5}}-1\right)$$

(2.4) $$g_{3}=\frac{m_{3}}{m_{4}}\frac{1}{N}\sum_{i=1}^{N}\langle \lambda_{i}-{1\rangle }^{2m_{4}}$$

where 〈 〉 represents the Macauley brackets, which indicates that the fibers do not bear compression loads (Buerzle and Mazza, 2013) and $m_{i}$ are model parameters. The parameter N represents the number of representative families and it must be an even number. The first invariant of the right Cauchy-Green deformation tensor is represented by $I_{1}$, while J is the volume ratio, and $\lambda_{i}$ is the fiber stretch (Buerzle and Mazza, 2013; Mauri et al., 2016):

(2.5) $$I_{1}=tr\left({\text{F}}^{T}\text{F}\right)$$

(2.6) J=detF

(2.7) $$\lambda_{i}=\left|\text{F}{\text{M}}_{i}\right|,i=1,2,\ldots ,N$$

The deformation gradient is represented by F and M represent the fiber directions in the reference configuration and it is defined as (Mauri et al., 2016):

(2.8) $${\text{M}}_{j}=cos\;\beta_{j}\;sin\;\theta_{j}{\text{e}}_{1}+sin\;\beta_{j}\;sin\;\theta_{j}{\text{e}}_{2}+cos\;\theta_{j}\text{N}$$

(2.9) $${\text{M}}_{j+N/2}=cos\;\beta_{j}\;sin\;\theta_{j}{\text{e}}_{1}+sin\;\beta_{j}\;sin\;\theta_{j}{\text{e}}_{2}-cos\;\theta_{j}\text{N}$$

(2.10) $$\beta_{j}=\frac{2\pi }{N}\left(j-\frac{3}{2}\right)$$

(2.11) $$\theta_{j}=\frac{\pi }{2}-\varphi$$

where φ represents a slight off-plane inclination and $j=1,2,\ldots ,\frac{N}{2}$. The Cauchy stress tensor σ is obtained from the derivatives of the strain energy density function with respect to the deformation tensor (Buerzle and Mazza, 2013). The membrane tension t in the current configuration was calculated through the following equation, where $h_{0}$ is the initial thickness of the membrane, and $\lambda_{1}$ and $\lambda_{2}$ are the longitudinal and lateral stretches, respectively (Buerzle and Mazza, 2013):

(2.12) $$\text{t}=\sigma \frac{h_{0}}{\lambda_{1}\lambda_{2}}$$

In total, eight parameters must be defined: $\mu_{0},\;q,\;m_{2},\;m_{3},\;m_{4},\;m_{5},\;N$ and φ.

The fetal membrane may be subjected to large deformations during biaxial mechanical tests. In this scenario, the $g_{3}$ function likely reaches considerable values, due to the exponential term $e^{qg}$. Consequently, the strain energy function will also reach large values, which may result in illogical values for the stress and Jacobian matrices. Therefore, a stability control was considered for the $g_{3}$ function, limiting its maximum value to a certain threshold. Every time the limit was surpassed, the iteration was restarted with a smaller value for the time increment. It is important to highlight that this control does not have any effect on the results. The control was implemented in Abaqus/Standard, where the user material subroutine was also developed.

#### 2.1.2. Amnion material parameter characterization

The values of the amnion constitutive parameters were set according to Table 1, provided by Mauri and Ehret (Mauri et al., 2016), except for the $\mu_{0}$ and φ parameters. The first was determined through trial and error, using a uniaxial finite element setup (subsection 2.1.2 a)). Inflation (subsection 2.1.2 b)) and puncture (subsection 2.1.2 c)) setups were also developed to validate $\mu_{0}$ and evaluate the prediction capabilities of the amnion model. The outcomes from these three numerical tests were compared to experimental data from the literature (Buerzle and Mazza, 2013; Oyen et al., 2004) in the results section. The parameter φ was set to 0 to exclude the impact of the fiber’s off-plane inclination.

##### (a) Uniaxial test

**Fig. 1.**
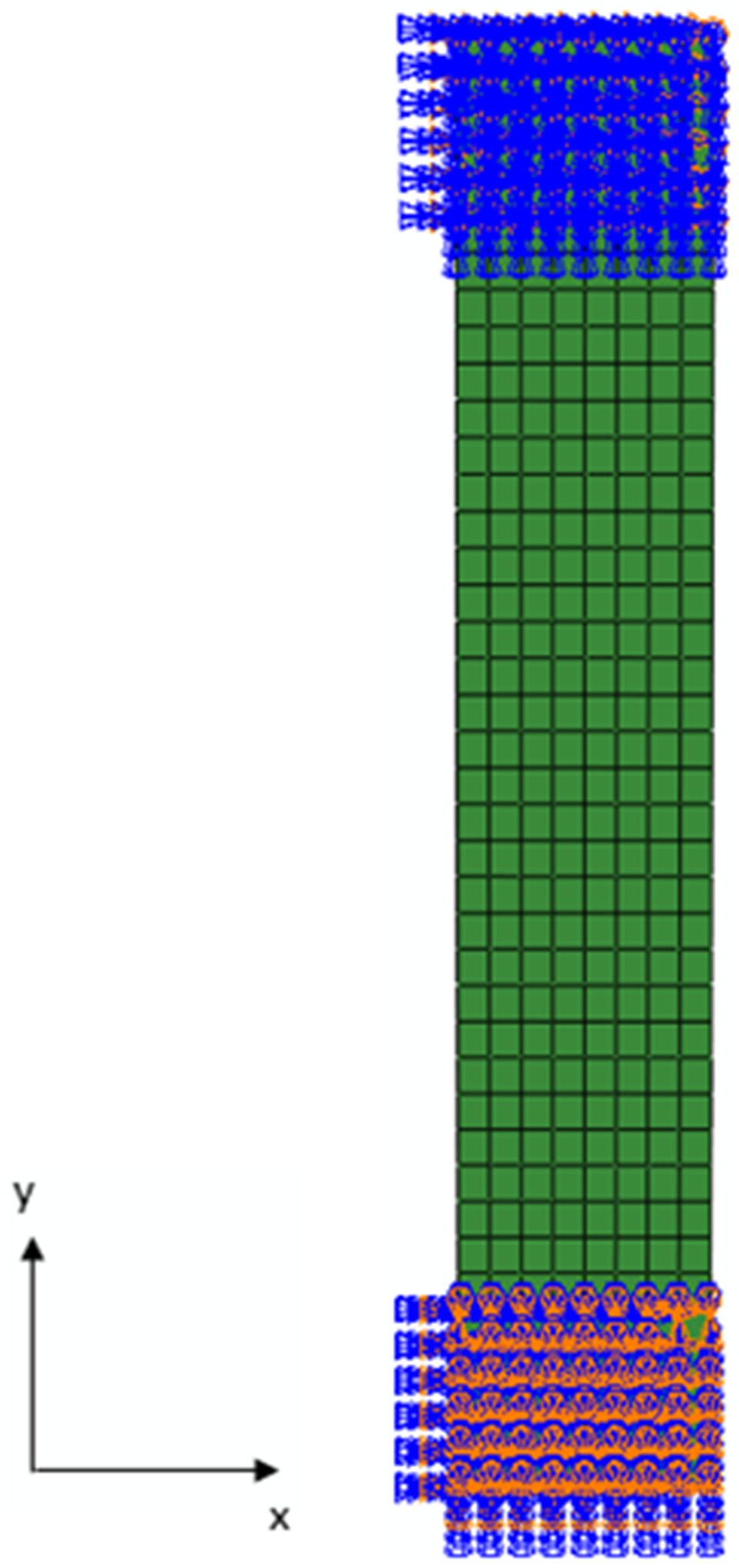
Finite element setup of a uniaxial test and respective boundary conditions.

To obtain $\mu_{0}$ through trial-error analysis, a finite element setup of a uniaxial mechanical test was created according to the experimental setup reported in Buerzle et al. (Buerzle and Mazza, 2013), discretizing a rectangular sample of dimensions 80 × 15 mm in 320 eight-node brick (C3D8) finite elements and 739 nodes, as illustrated in Fig. 1.

To simulate the mechanical conditions of the uniaxial test, the tissue corresponding to 10 mm measured from the bottom of the sample was restrained in all directions. The same restriction was applied for 10 mm measured from the top of the sample, except for the y-direction, where a displacement boundary condition was applied. Therefore, the sample starts from an initial free length of 60 mm. A displacement of 15 mm was applied in the upper region of the sample, following the positive direction of the y-axis.

Different uniaxial stretch-tension curves were obtained for different $\mu_{0}$ values until the numerical curve replicated almost perfectly the experimental curve, especially for smaller stretches (Buerzle and Mazza, 2013).

##### (b) Inflation test

**Fig. 2.**
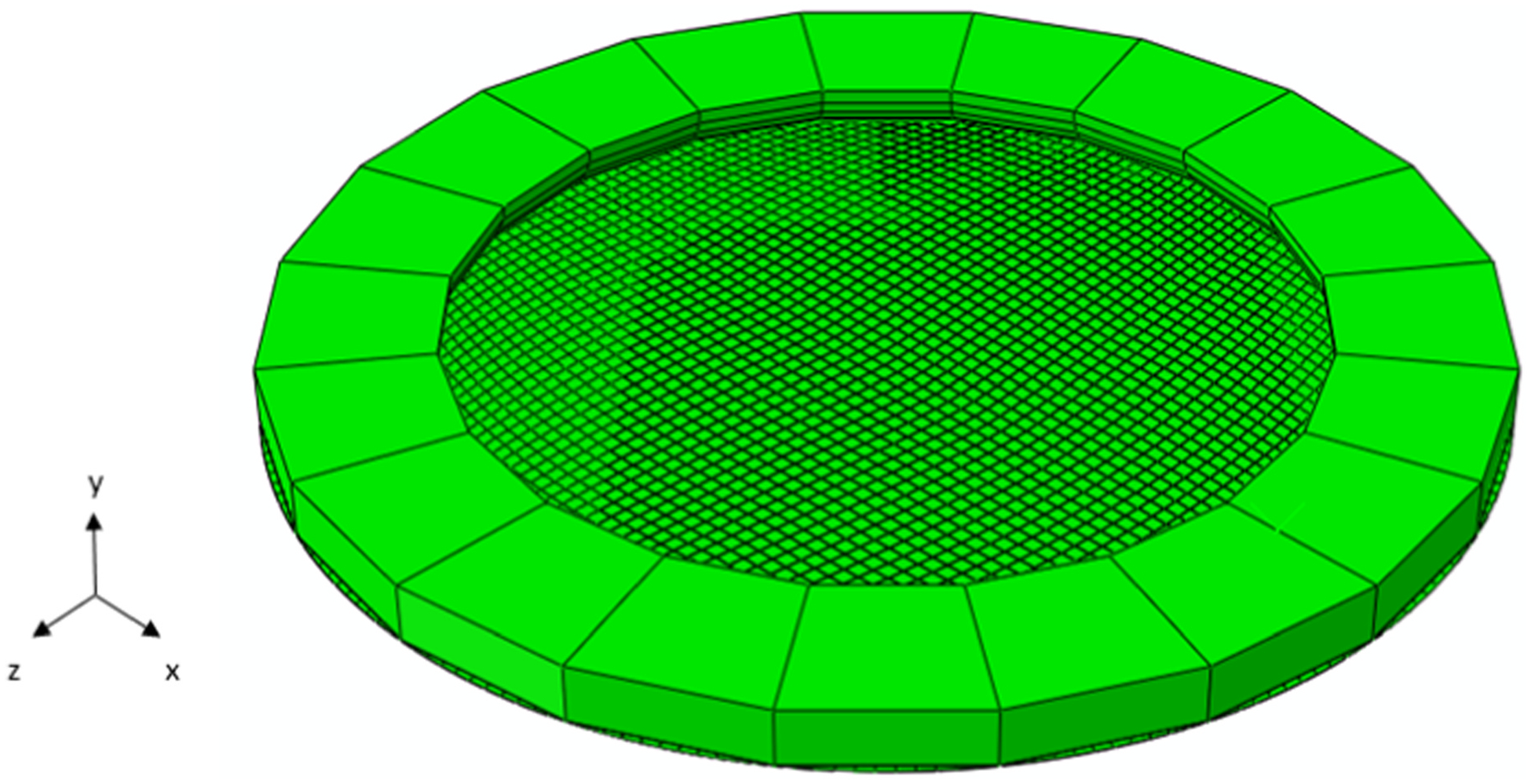
Finite element setup of an inflation mechanical test.

After obtaining $\mu_{0}$, an inflation setup was developed to evaluate the prediction capabilities of the amnion model, comprising the amniotic sample and a clamping ring (Buerzle and Mazza, 2013). To match the literature’s mechanical test, the sample was 70 mm in diameter and contained 4667 C3D8 finite elements and 9555 nodes (Fig. 2). The clamping ring was characterized as a rigid body with an inner diameter of 50 mm and an outer diameter of 70 mm.

The clamping ring was fixed and the nodes from the border of the amnion were restricted in all directions. The contact between the amnion and the clamping ring was modeled as surface-to-surface, with a high friction coefficient (0.9). A ramping pressure of 10 kPa distributed across the lower surface of the amnion was applied in the positive direction of the y-axis.

Following a similar procedure as the uniaxial test, the tension-stretch evolution from the model during an inflation test was also compared with averaged biaxial tension-stretch experimental data using amnion samples, maintaining the $\mu_{0}$ value obtained in the uniaxial test (Haller, 2013).

##### (c) Puncture test

**Fig. 3.**
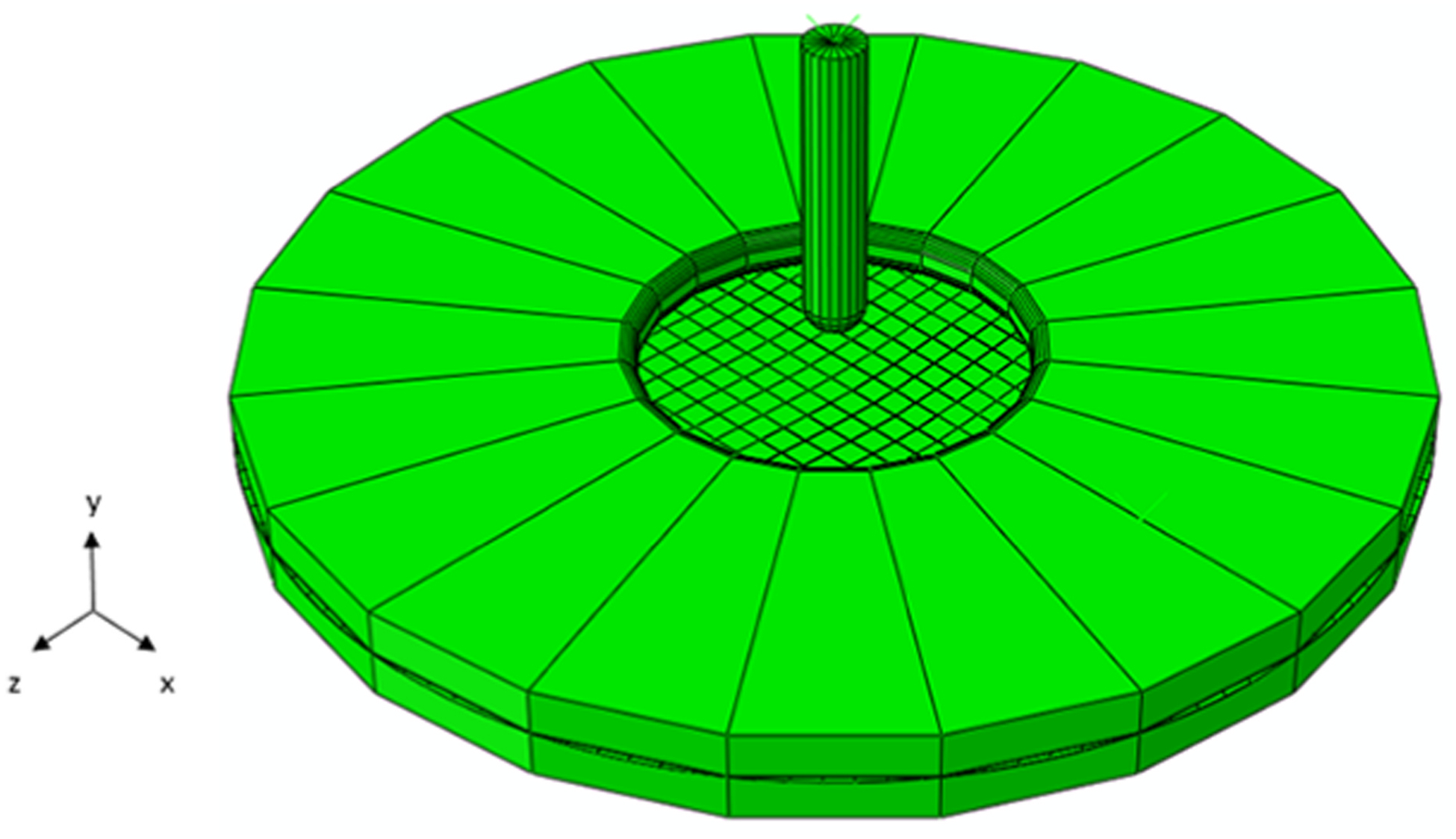
Finite element setup of a puncture mechanical test.

Similarly to the inflation test, a puncture setup was also developed to validate and verify the prediction capabilities of the amnion model, comprising the amniotic sample, an upper clamping ring, a lower clamping ring, and a puncture probe, according to Oyen et al. (2004). The sample was 60 mm in diameter and contained 1561 C3D8 finite elements and 3427 nodes. The clamping rings and the puncture probe were characterized as rigid bodies. The first had an inner diameter of 20 mm and an outer diameter of 60 mm, while the latter had 3.2 mm in diameter (Fig. 3).

The simulation of the puncture test was divided into three steps: (1) descent of the upper clamping ring, (2) application of a clamping force, and (3) descent of the puncture probe. Steps (1) and (2) represent the necessary preconditioning to perform the puncture test. Step (1) implied that the upper clamping had its movements restricted, except for the y-direction, and the lower clamping ring was fixed. Step (3) allowed the puncture to push the membrane 10–15 mm, a distance that usually causes the amnion to rupture (Oyen et al., 2004); in this step, the puncture probe was restricted in all directions, except for the y-direction; finally, the nodes from the membrane border were fixed. The contact between the amnion and the clamping rings was modeled as surface-to-surface, with a high friction coefficient (0.9), while the contact between the puncture and the membrane was modeled as frictionless contact.

The reaction force caused by the puncture probe was compared with the literature in the Results section, pushing the membrane 10–15 mm. This simulation helped validate the amnion model with $\mu_{0}$ set to the value obtained in the uniaxial test (Oyen et al., 2004).

#### 2.1.3. Chorion and decidua characterization

The chorion and decidua are less mechanically dominant than the amnion layer. Therefore, these layers were characterized as linear elastic materials, with parameters retrieved from the literature (Table 2). In this work, it was hypothesized that the chorion and the decidua shared similar mechanical properties since it is known that the smooth chorion becomes fused with the decidua, which may suggest that they are similar from a stiffness perspective. The respective Poisson’s coefficients were set to a value near 0.5 (Roohbakhshan et al., 2016; Verbruggen et al., 2017).

### 2.2. Multilayer fetal membrane model

**Fig. 4.**
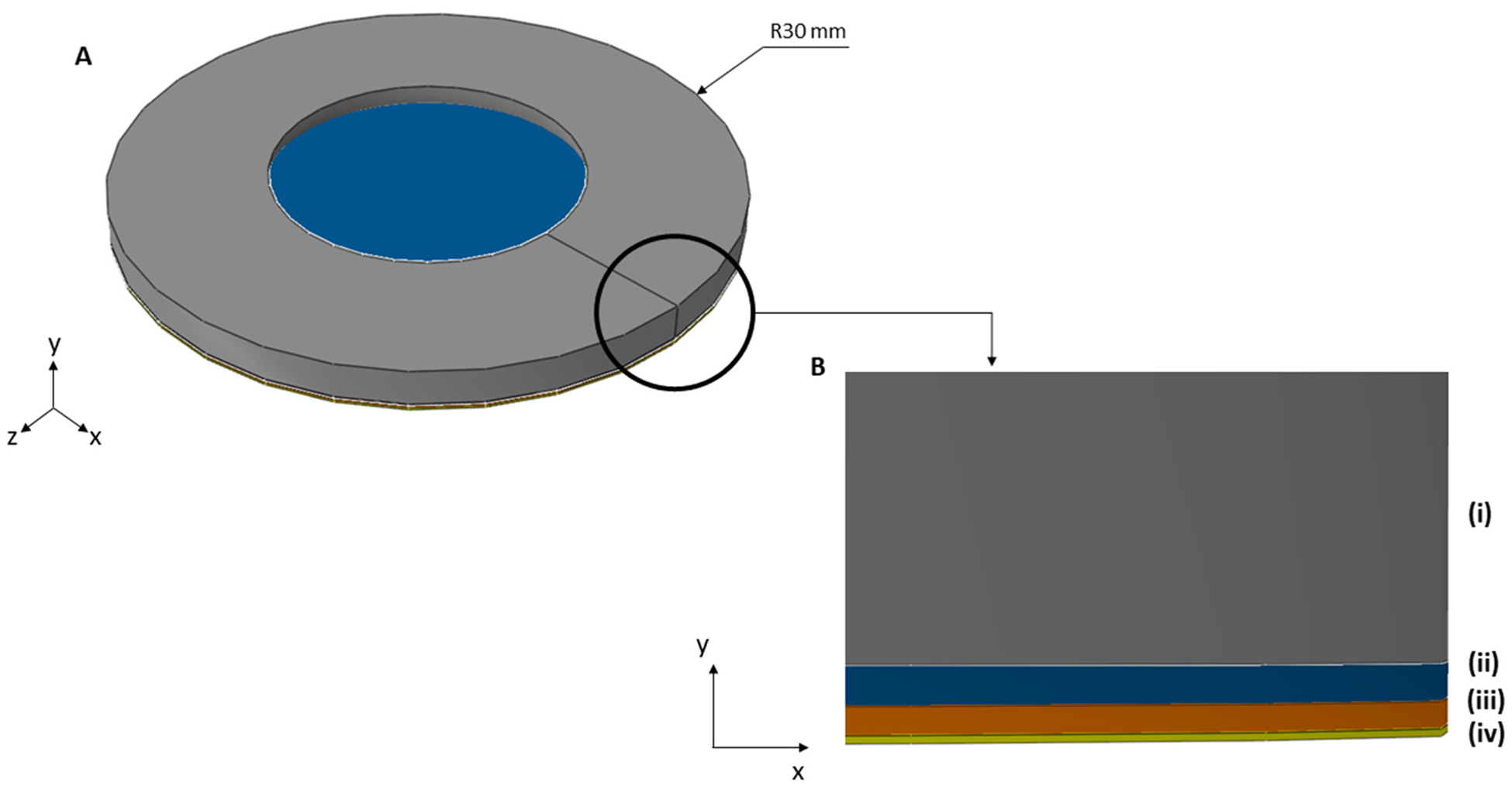
Finite element setup of the inflation mechanical test using the multilayer fetal membrane model; A: three-dimensional view of the model; B: detailed view of the model (grey: clamping ring (i); blue: part of the decidua (ii); orange: chorion (iii); yellow: amnion (iv)).

After modeling the amnion, chorion, and decidua, a multilayer fetal membrane structure was developed to capture the inflation response of the amnion-chorion-decidua layers. A finite element model of the inflation test presented by Samimi et al. was created (Samimi et al., 2023), as illustrated in Fig. 4.

Details on the mechanical tests are found in Samimi et al. (2023). Briefly, the fetal membrane samples were tested in a custom inflation mechanical test rig, where membrane pressure and deformation were measured simultaneously. Optical coherence tomography (OCT) and machine learning methods were developed to characterize the *ex vivo* properties of fetal membranes under dynamic loading. These were *in situ* mechanical tests, where the thickness of each layer was updated during inflation using OCT. The test was performed by laying the samples flat on a circular plate, with a 30 mm-diameter opening in the center and an external diameter of 60 mm. A clamping ring with the same dimensions was placed over the samples and secured with equally spaced bolts and nuts. A constant-flow syringe pump connected to an open water column at the base of the setup provided a nearly linear saline pressure ramp at a rate of 10 kPa per minute until rupture. A pre-inflation pressure value of approximately 0.2 kPa was applied before the test to eliminate the initial clamped membrane’s slack state. Eighteen 6 cm diameter peri-placental samples from unlabored elective cesarian sections collected between 37 and 41 weeks of gestation were selected for this work.

The multilayer fetal membrane model developed under an inflation setup (Fig. 4) comprises the following structure: (i) clamping ring, (ii) part of the decidua, (iii) chorion, and (iv) amnion. The spongy layer was modeled as a contact condition between the amnion and the chorion and not as a physical layer since it only allows the amnion to slide on the underlying chorion. The spongy layer easily disintegrates during the separation process for mechanical testing, making it difficult to characterize it in isolation.

The amnion had a diameter of 60 mm. Its thickness was set to the average value of all selected samples’ initial thicknesses reported in the experimental dataset (Table 3). The amnion was mechanically characterized by the modified version of the Buerzle-Mazza constitutive model, setting the respective constitutive parameters to the values reported in Table 1. Once the solid model was constructed, the amnion was meshed with 3422 C3D8 elements and 7034 nodes.

The chorion and the decidua also had a diameter of 60 mm and the respective thicknesses were set as described for the amnion (Table 3). These layers were characterized as elastic linear materials, (Table 2). The chorion and decidua were both meshed with 3422 C3D8 elements and 7034 nodes.

The clamping ring had an inner diameter of 30 mm and an outer diameter of 60 mm. A fillet edge was considered to avoid cutting the membrane during the mechanical test. This structure was modeled as a rigid body.

The contact condition between the amnion and the chorion was modeled as a surface-to-surface contact with a friction coefficient of 0.5, playing the role of the spongy layer. The chorion and the decidua were tied, representing the fusion process mentioned before. Finally, the contact between the decidua and the clamping ring was modeled as a surface-to-surface contact, with a high value of friction coefficient (0.9). Concerning the boundary conditions, all the border nodes from the three layers were restricted in all directions and the clamping ring was fixed.

### 2.3. Simulation steps of the inflation mechanical test

The fetal membrane samples were first submitted to a pre-inflation state, which corresponded to a slightly distended condition of the membrane, and it was completely different from the slack state of the initially clamped membrane. This procedure was performed to obtain a repeatable definition of the reference configuration for the analysis of the experiments (Myers et al., 2010).

Therefore, the inflation simulation was divided into two main steps: (i) pre-inflation, and (ii) inflation-to-failure. In step (i), a pre-inflation pressure ranging between 0.1 and 0.3 kPa was applied, to reach the pre-inflation state, while in step (ii) a ramping inflation pressure ranging between 10 and 20 kPa was considered. The pre-inflation pressure and the ramping inflation pressures were sequentially applied across the inferior surface of the amnion layer, in the positive direction of the y-axis (Fig. 4).

## 3. Results

The results are divided into three parts: (3.1) $\mu_{0}$ for the amnion material model is obtained by analyzing uniaxial behavior, and the prediction capabilities of the material model are analyzed under inflation and puncture mechanical setups, (3.2) the apex displacement of the multilayer fetal membranes model (anisotropic amnion) is compared to the experimental apex displacement and to a case where the multilayer model comprises an elastic amnion material characterization (Verbruggen et al., 2017), (3.3) for the same inflation simulation, the mechanical response of the amnion and the chorion is analyzed.

### 3.1. Amnion material model calibration and validation

**Fig. 5.**
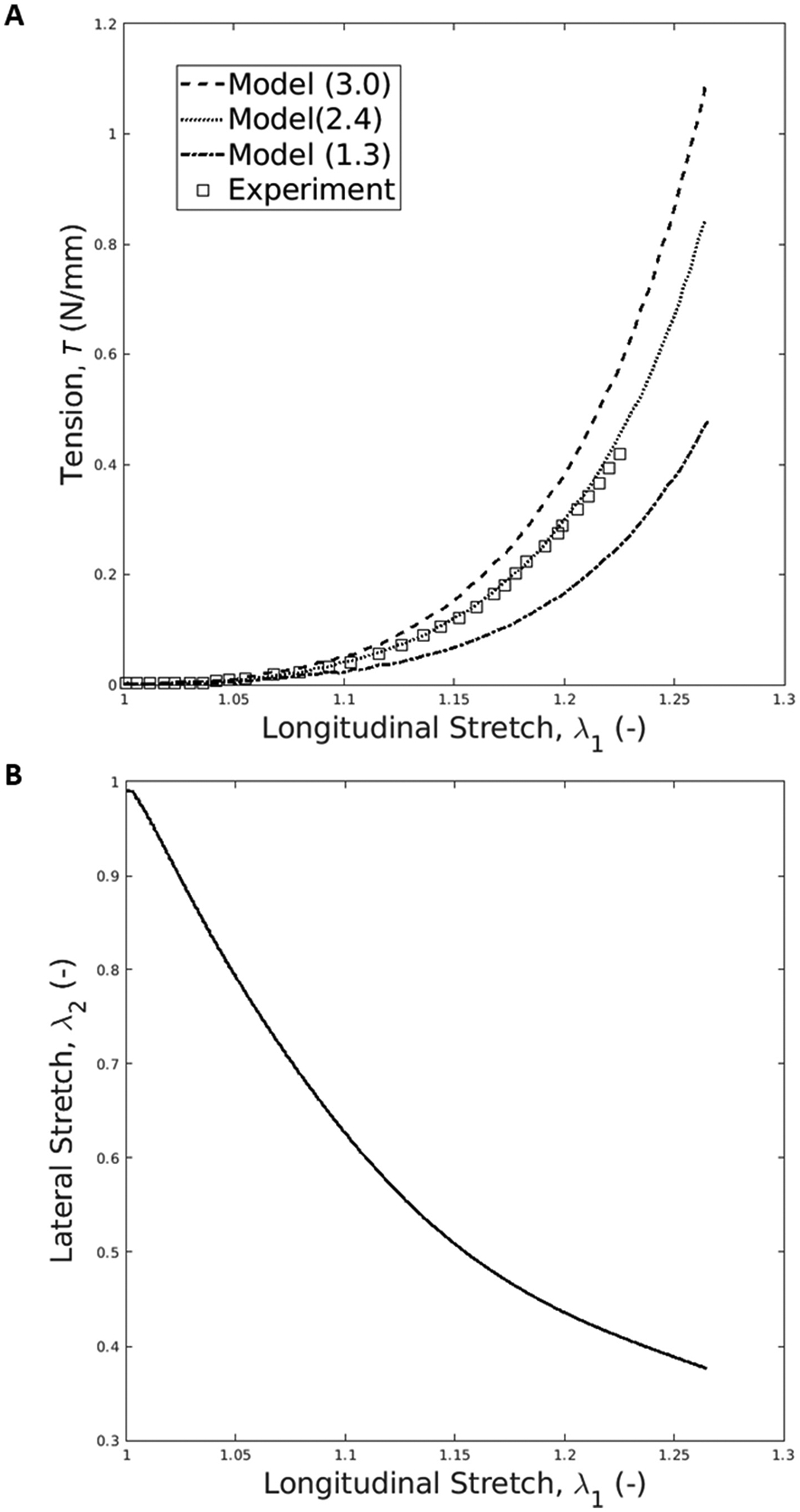
(A) Trial-error analysis to obtain the $\mu_{0}$ parameter; $\mu_{0}$ is set to several values (only three are here illustrated) and the respective tension-stretch curves are compared with experimental data; the best match is obtained when $\mu_{0}$ is set to 2.4 MPa; (B) lateral stretch as a function of longitudinal stretch in our amnion model during the uniaxial test.

The nonlinear behavior of the amnion is featured in the model and experimental response to uniaxial extension, where the longitudinal stretch $\left(\lambda_{1}\right)$ – tension (T) curves exhibit small stiffness values for smaller stretches, followed by a transition to a larger stiffness (Fig. 5). The material parameter $\mu_{0}$ is set to several values and the best match between the model’s stretch-tension curve and the experimental data occurs when $\mu_{0}$ is set to 2.4 MPa (Fig. 5A), especially for smaller deformations. For this value, the mean-squared error for 5% of longitudinal stretch is equal to 0.0000040, for 20% is equal to 0.00023, and for 100% is equal to 0.00030. Lastly, the relation between lateral deformation and longitudinal contraction is analyzed (Fig. 5B). In uniaxial extension, the lateral stretch $\left(\lambda_{2}\right)$ evolution shows that the amnion layer has a remarkable lateral contraction as the membrane stretches longitudinally.

**Fig. 6.**
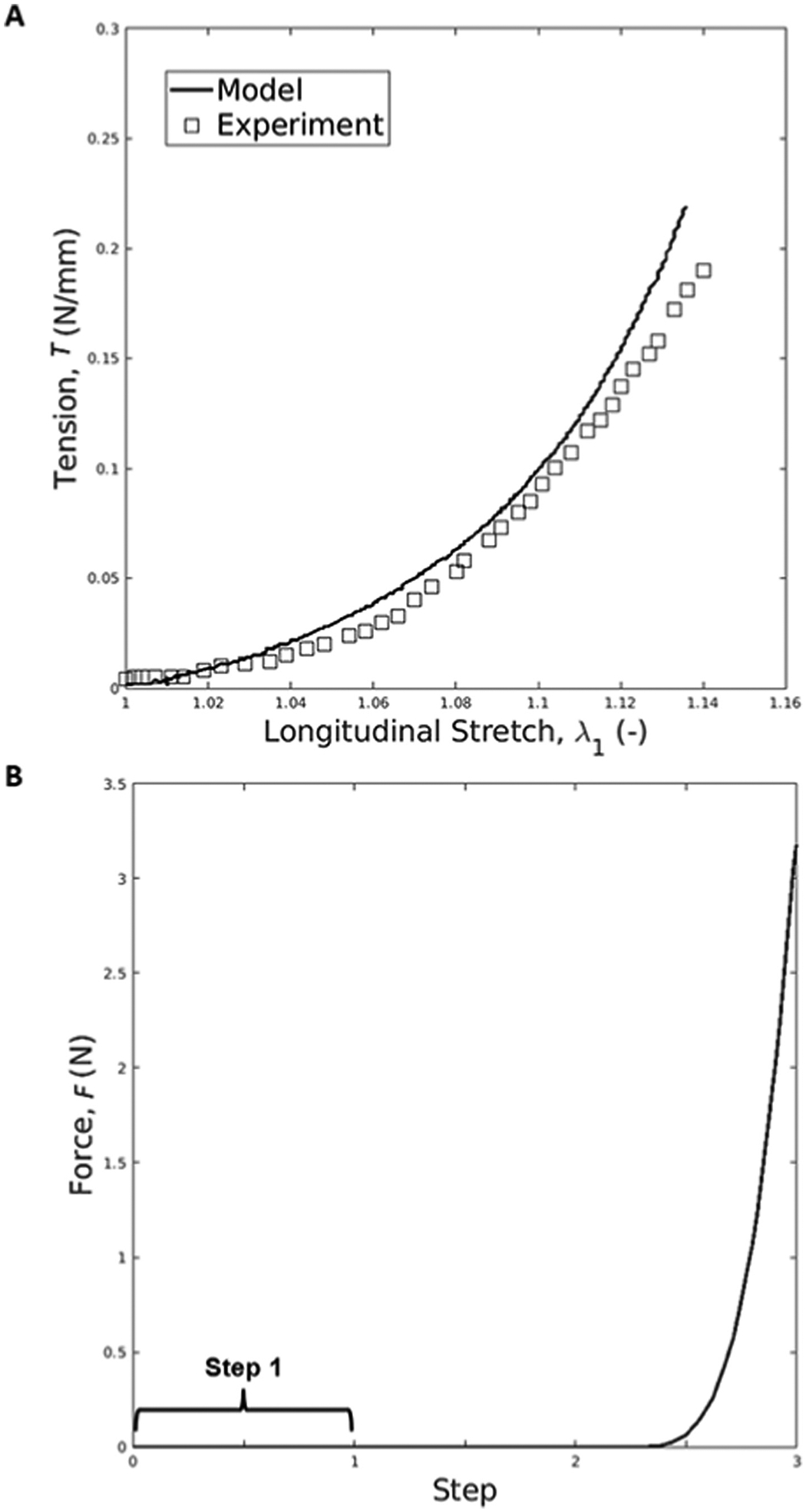
(A) Evolution of tension at the apex region of the amnion as a function of the longitudinal stretch during the inflation test.; (B) Evolution of the force caused by the probe during the puncture test.

The amnion material parameters are validated by comparing computational results with published experimental values. For smaller values of stretches in inflation, the model curve matches the experimental data (Fig. 6A). For larger stretch values, the model and experimental curves exhibit a slight deviation, which is also observed in previous works (Buerzle and Mazza, 2013). Regarding the puncture test, the force caused by the probe (F) increases as it pushes the membrane, registering a maximum value of 3.18N when the probe descends about 11 mm (Fig. 6B), similar to what is verified in the work of Oyen et al. (2004).

### 3.2. Multilayer fetal membrane model

**Fig. 7.**
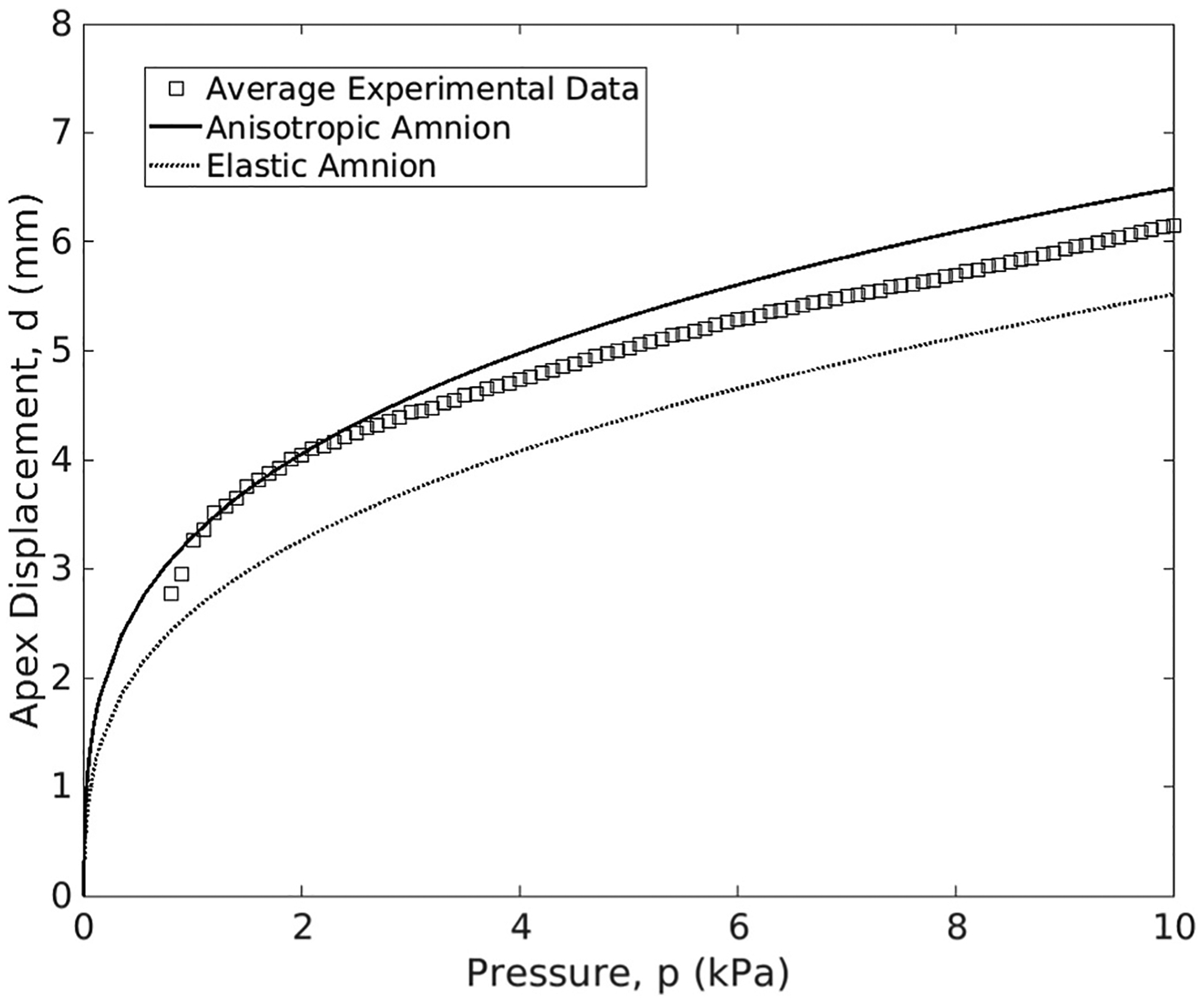
Comparison of the multilayer fetal membrane model (anisotropic and elastic amnion) with experimental data from Samimi et al. (Samimi et al., 2023); the elastic amnion is characterized according to Verbruggen et al. (Verbruggen et al., 2017).

The evolution of the membrane’s apex displacement (*d*) for the multilayer finite element model (anisotropic and elastic amnion) and the experimental test is represented in Fig. 7. The experimental curve is obtained by averaging the apex displacements of the eighteen C-section fetal membrane samples from the peri-placental region, after performing a data interpolation to obtain a common grid of points along the pressure axis. The common pressure values across all eighteen samples range between 1 kPa and 10 kPa, approximately.

For all cases, the apex displacements tend to increase faster during the initial stage of the inflation simulation, which corresponds to the pre-inflation step, where the membrane is in a slack state. As pressure (p) increases, the curves start to flatten since the membrane is offering more resistance. Therefore, the apex displacement variation tends to decrease throughout the simulation. For smaller pressures (<2 kPa), the apex displacement of the multilayer model containing the anisotropic amnion overlaps the experimental data, while the model containing the elastic amnion leads to considerably smaller apex displacements. For larger pressures, the relative error at 10 kPa, $\xi_{r}$, verified between the experimental data and the anisotropic amnion model is 5.0% (equation (3.1)) and 11.0% for the elastic amnion model (equation (3.2)). Moreover, the standard deviation was calculated to measure how dispersed the data was compared to the mean curve. A value of 1.764 was calculated, indicating that the data is spread out around the mean curve.


(3.1) $$\xi_{r\_\text{anisotopic}}\left(\text{\%}\right)=\frac{\text{Simulation}\;\text{Value}-\text{Experimental}\;\text{Value}}{\text{Experimental}\;\text{Value}}=\frac{6.49-6.18}{6.18}=5.0\text{\%}$$

(3.2) $$\xi_{r\_\text{elastic}}\left(\text{\%}\right)=\frac{5.50-6.18}{6.18}=11.0\text{\%}$$

### 3.3. Stress distribution and thickness variation in the amnion and chorion layers

**Fig. 8.**
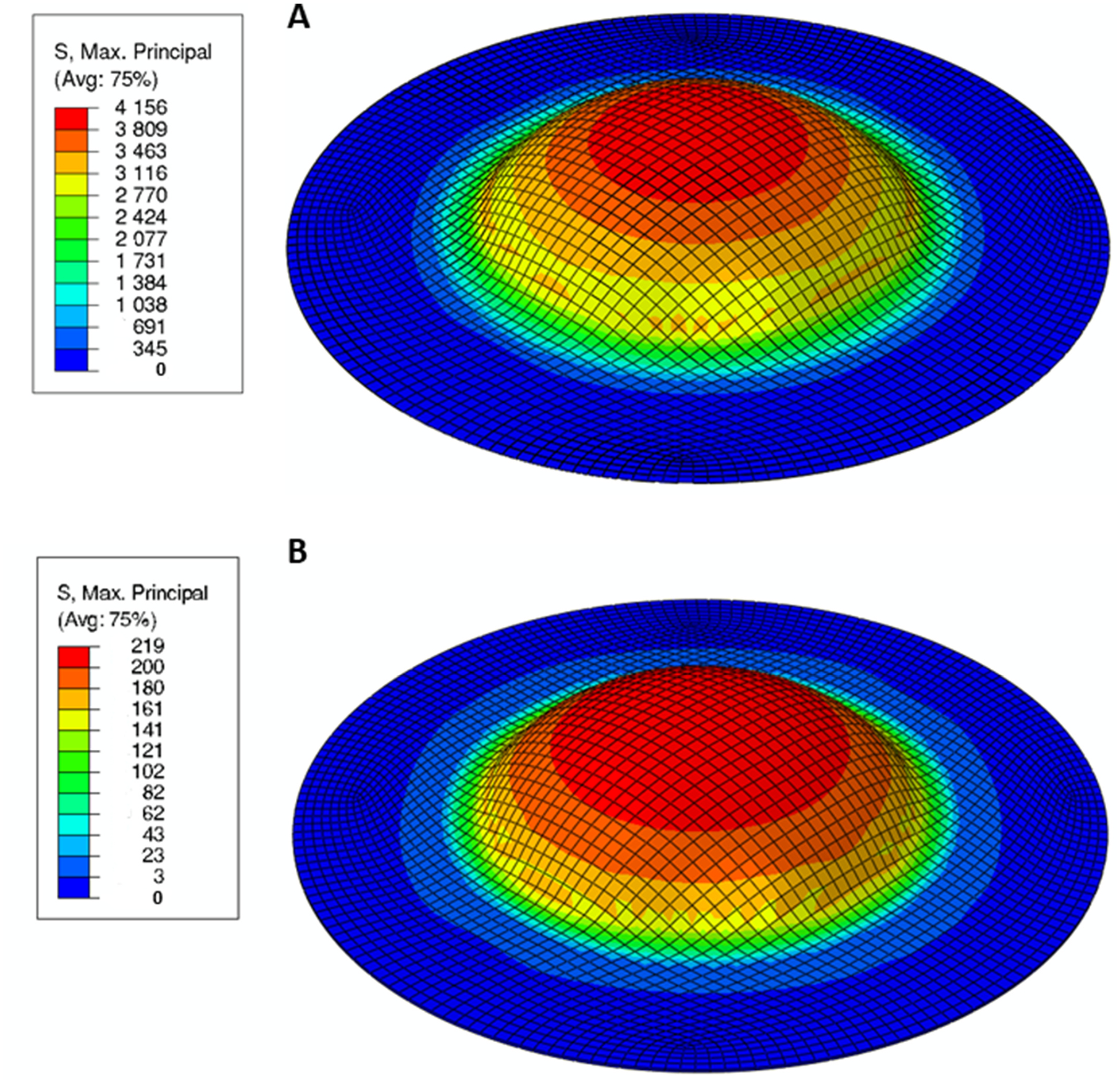
Maximum principal stress distribution in the amnion (A) and chorion (B) layers at the end of the simulation of the inflation mechanical test (units: kPa).

**Fig. 9.**
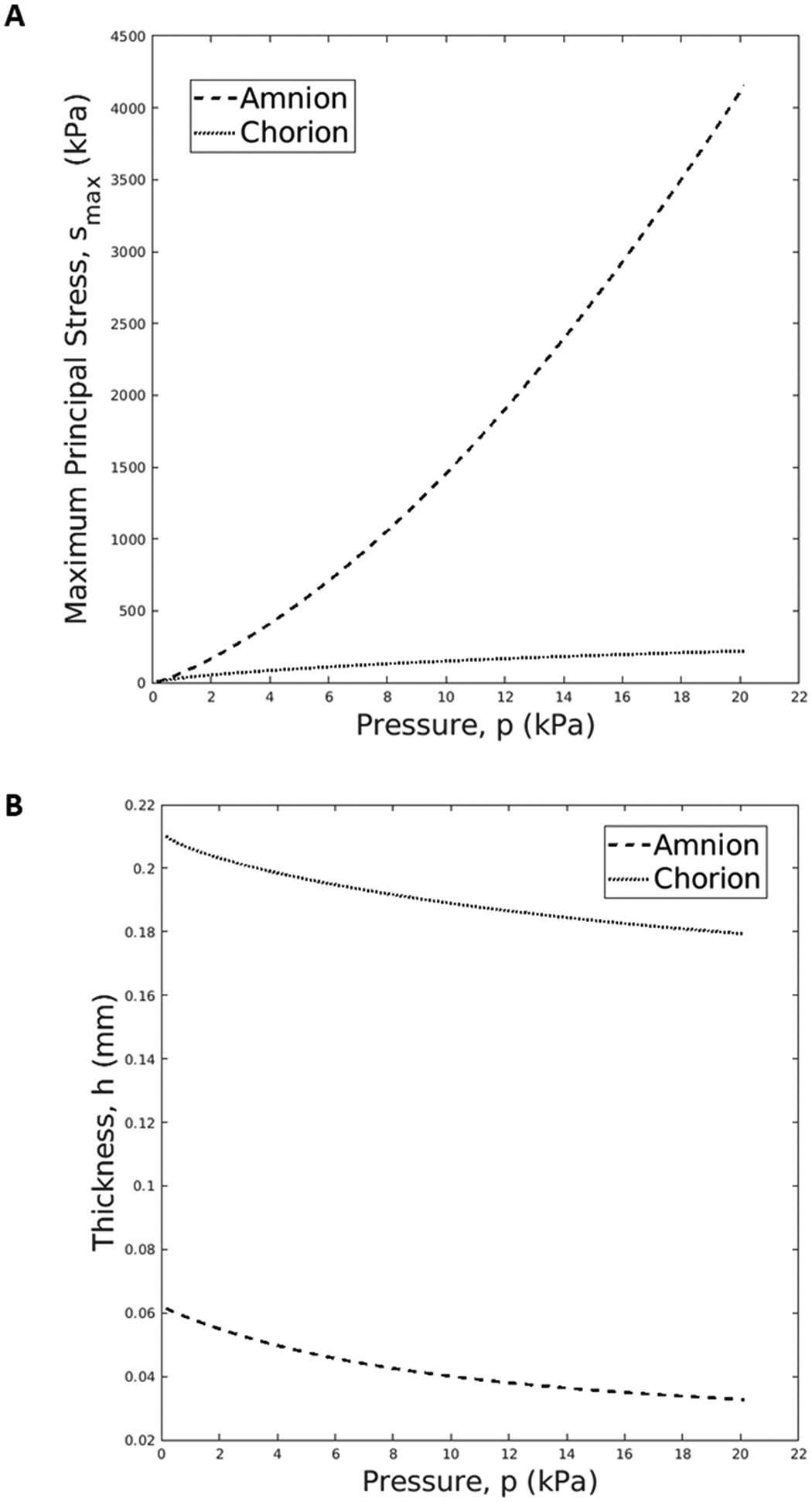
(A) Maximum principal stress evolution in the amnion and chorion layers throughout the simulation of the inflation mechanical test; (B) Thickness evolution of the amnion and chorion throughout the inflation simulation.

The maximum principal stress ($s_{\text{max}}$) distribution in the amnion and chorion layers at the end of the inflation simulation is illustrated in Fig. 8, and the maximum principal stress evolution throughout the inflation test at the apex region of each fetal membrane layer is represented in Fig. 9A. At the end of the inflation test, where an apex displacement of 7.8 mm is verified, the amnion layer exhibits a maximum principal stress value of 4156 kPa at the apex region, while the chorion only exhibits a value of 219 kPa (Fig. 8). It is also important to highlight that the amnion is subjected to much larger stresses than the chorion throughout the entire mechanical simulation of the inflation test (Fig. 9A). There is a thickness (h) reduction in the amnion and the chorion throughout the simulation at the apex region (Fig. 9B): the chorion has a reduction of 15% while the amnion has a reduction of 47%.

## 4. Discussion

Term birth involves a set of interrelated biological and biomechanical events, including cervical remodeling and deformation, rupture of the fetal membrane (ROM), and coordinated uterine contractions (Vink and Feltovich, 2016). These events may happen too soon in gestation, such that the mechanical failure of the soft tissues supporting the fetus can lead to spontaneous preterm birth (Vink and Feltovich, 2016). To support pregnancy, there is a complex biomechanical interaction between the cervix, uterus, and fetal membranes. This interaction and how each tissue contributes to load-bearing remains to be understood entirely. In this context, numerical methods are an essential tool to overcome technical and ethical obstacles associated with understanding the biomechanical roles of the soft tissues supporting the fetus.

In this work, a multilayer fetal membrane model, including the amnion, chorion, and maternal decidua, was developed based on robust experimental data from the literature. The mechanically dominant amnion was characterized as an anisotropic nonlinear material to capture its fiber response. This represents an advantage over existing numerical models, where the mechanical role of the amnion fibers can be explored. This multilayer model can be implemented in more complex numerical uterine models of the gravid uterus to investigate the interaction between the fetal membrane, uterus, and cervix, and how the fetal membrane layers interact with the uterus to prevent large deformations for the membrane and the cervix.

### 4.1. Amnion material model

It is hypothesized that collagen fibers play an important role in the mechanical response of the amnion. To understand the extent of this role, a fiber-based hyperelastic constitutive model for the amnion was developed and validated against a set of experimental data (Figs. 5 and 6). The amnion model was developed by Buerzle and Mazza, based on a theory developed by Rubin and Bodner, where the nonlinear dissipative response of soft tissue as the amnion was studied (Rubin and Bodner, 2002). Since soft tissue acts as a complex composite structure, where families of fibers are embedded in an isotropic matrix, the constitutive equations presented by Rubin and Bodner comprise a purely elastic component and a dissipative component (Rubin and Bodner, 2002). The model developed by Buerzle and Mazza only accounts for the elastic part of the Rubin-Bodner formulation, since their experimental data does not provide any information on the time and history dependence of the amnion layer (Buerzle and Mazza, 2013).

The fibers embedded in the amnion layer exhibit resistance to elongation, leading to their rotation (Buerzle and Mazza, 2013). These phenomena will reduce their angle with respect to the direction of tension, leading to large lateral contraction, which is in line with the work of Kabla and Mahadevan concerning the nonlinear mechanics of soft fibrous networks (Buerzle and Mazza, 2013; Kabla and Mahadevan, 2007). These mechanical characteristics align with Fig. 5B, where the amnion sample underwent significant lateral contraction compared to the longitudinal stretch, indicating that the amnion possesses a high Poisson’s ratio.

The prediction capabilities of the amnion model were also evaluated in Fig. 6A, where an inflation test was performed. As the figure suggests, the model predicted the tension evolution, especially for longitudinal stretch values smaller than 1.15. For larger values, the finite element curve diverged from the experimental curve, a tendency also verified by Buerzle and Mazza et al. (Buerzle and Mazza, 2013). Lastly, the amnion model was assessed with a biaxial puncture test, comparing the values of the pushing force to those reported in the literature for specific displacements (Oyen et al., 2004). According to Oyen et al. the puncture force for peri-placental C-section amnion samples varies between 2.81 N and 3.55N (Oyen et al., 2004). The puncture test simulation resulted in a maximum force of 3.18 N (Fig. 6B), following the same conditions as Oyen et al. This means that the amnion model can predict the mechanical behavior of the amnion during the loading portion of a puncture test.

### 4.2. Multi-layer fetal membranes model

Once the amnion was characterized, as well as the chorion and the decidua, the multilayer fetal membrane model was developed under an inflation setup. The inflation simulation was divided into two steps: (i) pre-inflation and (ii) inflation. The pre-inflation step was performed to identify the “zero stress state” of the membrane, which is difficult to obtain due to its low initial stiffness (Gao and Desai, 2010). Therefore, it is common to apply a low force, two orders smaller than the maximum force (Buerzle and Mazza, 2013). In this work, a pre-inflation pressure raging between 0.15 kPa and 0.3 kPa was considered, fulfilling this condition.

The validation of the multilayer fetal membrane model was performed in three phases: (i) comparing the numerical and experimental inflation apex displacements (Fig. 7), (ii) comparing the numerical maximum principal stress values observed in the amnion and chorion with the respective physiological stress ranges reported in the literature (Figs. 8 and 9A), and (iii) comparing the thickness variation of the chorion throughout the inflation simulation with the experimental data (Fig. 9B). The apex displacement of the multilayer fetal membrane model (anisotropic amnion) captured the evolution of the experimental displacement for low-pressure values and exhibited a relative error of 5% at 10 kPa (Fig. 7). This forward prediction of the model to newly published inflation experimental data (Samimi et al., 2023) represents an achievement from a computational perspective since the material model parameters were calibrated on different sets of mechanical experiments in the literature.

The fiber-based multi-layer model found that the amnion is subjected to larger stress levels, similar to the results found in a previous work by Verbruggen et al. In that work, a bilayer fetal membrane model was developed, using elastic linear mechanical properties to define each layer (Verbruggen et al., 2017). It was found that the amnion was subjected to much larger maximum principal stresses throughout all gestation ages compared to the chorion (Verbruggen et al., 2017). It was also observed that a maximum stress value of 989 kPa in the amnion and a maximum value of 145 kPa in the chorion at 40 weeks of gestation when a pressure of 6.7 kPa was considered. Some of these observations are in line with Figs. 8 and 9A, where the amnion exhibited much larger maximum principal stresses than the chorion during the entire simulation of the inflation test. This means that the amnion is the dominant layer of the fetal membranes, withstanding the loads occurring throughout gestation (Buerzle and Mazza, 2013). Moreover, for a pressure of 6.7 kPa, our model predicted a stress value of 830 kPa in the amnion and 117 kPa in the chorion (Fig. 9A). Despite sharing the same order of magnitude, these values differ a little from previous works, probably due to the nonlinear behavior of the amnion considered in our layered model. Lastly, according to Fig. 9B, the chorion exhibited a decrease in thickness of 15%, which is in line with experimental data (Samimi et al., 2023). For the amnion layer, nothing can be concluded since its initial thickness is too small to be tracked accurately during mechanical experimenting.

### 4.3. Mechanical assumptions

Some assumptions were considered in this work: (i) a friction coefficient of 0.5 was applied in the interface between the amnion and the chorion, and (ii) the off-plane angle of the collagen fibers in the amnion was set to 0°.

The impact of different interfaces between the amnion and the chorion was investigated and it was verified that no differences were found in terms of maximum principal stress and the membrane’s apex displacement for friction and tie contacts. However, when a frictionless contact was applied, the maximum principal stress decreased in the amnion (1150 kPa compared to 1440 kPa at a pressure of 10 kPa), and it increased in the chorion (165 kPa compared to 150 kPa at the same pressure) in comparison with the other two interfaces. In terms of the membrane’s apex displacement, a slight increase was verified: 6.9 mm for frictionless contact and 6.5 mm for friction and tie contacts at a pressure of 10 kPa.

Moreover, the impact of different fiber off-plane angles in the amnion was also investigated: for larger pressure values, increasing the off-plane angle from 0° to 10° led to larger maximum principal stresses (1830 kPa compared to 1440 kPa at a pressure of 10 kPa) and larger thickness reduction (0.04 mm compared to 0.02 at a pressure of 10 kPa) in the amnion, despite leading to similar evolutions throughout the simulations. Larger angles led to different evolutions in terms of stress and thickness variations, which highlights the impact of the off-plane angle on fetal membranes’ biomechanics.

### 4.4. Limitations

There are limitations to this work associated with the modeling assumptions. First, the following modeling simplifications have been made: (i) the nonlinear behavior of the chorion and the decidua was not considered in the layered model, since it is particularly difficult to isolate these layers for experimental testing, (ii) the spongy layer was modeled as a mechanical contact between the amnion and the chorion and not as a physical layer, and (iii) the time-dependent material response was not considered. Additionally, the fibers in the amnion were assumed to have an affine deformation with the total solid continuum. Evidence from discrete fiber computational models shows that the non-affine motion of the fibers protects against membrane rupture (Koh and Oyen, 2015). The material modeling in this work did not explore fracture mechanics, nor the critical mechanical role of the fibers in preventing rupture. Another point that must be highlighted is the fact that the initial stress state of the fetal membrane at the beginning of the inflation mechanical test is not stress-free. It is difficult to experimentally replicate the existing conditions within the pregnant uterus, and it is also difficult to precisely detect the stress-free configuration of the membrane free of membrane wrinkles. The effect of small pre-stress in the membrane may be investigated in future works through a sensitivity analysis.

## 5. Conclusions

A multilayer fetal membrane model comprising an anisotropic nonlinear amnion was developed and validated using experimental data and information reported in the literature. The amnion was characterized as an affine continuum fiber-based model, while the chorion and maternal decidua were described as elastic linear materials. Results showed that this multilayer model could capture the overall mechanical behavior of the fetal membrane and the respective layers, obtaining good agreement with experimental data and information reported in the literature. This layered model can be integrated into larger models of the fetal membrane, uterus, and cervix to study the complex interaction between these structures in term and premature pregnancies.

## Figures and Tables

**Table 1:** Parameters of the modified Buerzle-Mazza constitutive model, according to Ehret, except for $\mu_{0}$ and φ (Mauri et al., 2016); all parameters are dimensionless.

Parameter	Value
q	2.96
$m_{2}$	0.00228
$m_{3}$	41.12
$m_{4}$	1.27
$m_{5}$	0.463
N	32
φ	0°

**Table 2:** Elastic linear properties of the chorion and the decidua layers (Ophir et al., 2002; Oxlund et al., 1990; Roohbakhshan et al., 2016; Verbruggen et al., 2017).

	Young’s Modulus, E [MPa]	Poisson’s Ratio, v
**Chorion**	1	0.4–0.5
**Decidua**	1	0.4–0.5

**Table 3:** Amnion, chorion, and decidua thicknesses.

	Thickness [mm]
**Amnion**	0.062
**Chorion**	0.217
**Decidua**	0.311

## Data Availability

The authors do not have permission to share data.

## Abbreviations

ROM: rupture of the fetal membranes
PPROM: preterm pre-labor rupture of the fetal membranes
OCT: Optical coherence tomography
